# Quercetin Potentiates the NGF-Induced Effects in Cultured PC 12 Cells: Identification by HerboChips Showing a Binding with NGF

**DOI:** 10.1155/2018/1502457

**Published:** 2018-02-28

**Authors:** Gallant K. L. Chan, Winnie W. H. Hu, Zoey X. Zheng, M. Huang, Yan X. Y. Lin, Caroline Y. Wang, Amy G. W. Gong, X. Y. Yang, Karl W. K. Tsim, Tina T. X. Dong

**Affiliations:** ^1^Shenzhen Research Institute, The Hong Kong University of Science and Technology, Shenzhen 518057, China; ^2^Division of Life Science and Center for Chinese Medicine, The Hong Kong University of Science and Technology, Clear Water Bay, Hong Kong; ^3^YNBY Lab Inc., No. 51, Xi-Ba Rd., Kunming, Yunnan Province 650032, China

## Abstract

Dementia is a persistent disorder of the mental processes and is strongly related to depression. However, the performance of current antidepression medicine is far from satisfactory. Herbal extract provides an excellent source to identify compounds for possible drug development against depression. Here, HerboChips were employed to search herbal compounds that could bind nerve growth factor (NGF). By screening over 500 types of herbal extracts, the water extract of Ginkgo Folium, the leaf of* Ginkgo biloba*, showed a strong binding to NGF. The herbal fractions showing NGF binding were further isolated and enriched. By using LC-MS/MS analysis, one of the NGF binding fractions was enriched, which was further identified as quercetin, a major flavonoid in Ginkgo Folium. Quercetin, similar to Ginkgo Folium extract, could enhance the effect of NGF in cultured PC 12 cells, including potentiation of neurite outgrowth and phosphorylation of Erk-1/2. This is the first report of discovering an NGF binding compound by using HerboChips from herbal extracts, which could be further developed for antidepression application.

## 1. Introduction

Dementia is a commonly mental problem found in elderly population today [1]. The symptoms of dementia included difficulties in thinking, interacting, and communication [2]. Depression, named as major depressive disorder (MDD), is a psychological and mental problem usually found in the patient of dementia [3]. Moreover, depression is especially common among those over 65 years of age and increases in frequency with age beyond this age [4, 5]. The signature of depression includes low self-esteem, loss of interest in normally delighted activities, inactive, and pain without an obvious cause [6]. About 2–7% of adults with major depression could result in suicide [7]. Today, there is neither preventive medication nor highly effective drugs for major depression [8].

Chinese medicine (CM) is renounced for treating diseases and relieving unpleasant symptoms especially for those related to aging problems [9–11]. However, the correlations between active ingredients and efficacies of those CM are poorly understood. In our previous reports, we have demonstrated a high-throughput screening platform, namely, HerboChips [12]. In brief, the extracts from different CM were separately by HPLC system: those separated fractions were then printed on an activated silico/plastic surface. On the other hand, the key regulator/signature for depression (e.g., nerve growth factor (NGF)) was labelled with biotin. After the reactions between the biotinylated regulator and different surfaces with immobilized fractions of CM extracts, a molecular reporter constructed by binding streptavidin with a fluorescence dye (e.g., Cy3 or Cy5) together was applied. Binding affinity between biotinylated NGF on specific CM was expected whenever a positive signal was shown after scanning. The CM with positive scanning result was submitted for biological evaluation using different cell assays. Eventual, the platforms successfully sought out potential CM candidates for possible development of antidepression drugs [12].

The extract of* Ginkgo biloba *leave, that is, Ginkgo Folium, was one of those many CM's extracts found with binding affinity to NGF as well as efficacies in antidepression functions [12]. Ginkgo Folium is one of the most well studied herbs in CM. The extract of Ginkgo Folium was rich in various bioactive compounds, including phenolic acids, proanthocyanidins, flavonoid glycosides, for example, myricetin, kaempferol, isorhamnetin, quercetin, and terpene trilactones, ginkgolides, and bilobalides [13, 14]. The identities of those bioactive compounds in Ginkgo Folium were well established. However, the NGF binding compounds have not been identified. Here, we demonstrated an application of HerboChips in searching NGF binding compounds from CM extracts, and Ginkgo Folium was shown to contain such a binding.

## 2. Materials and Methods

### 2.1. Chemicals and Herbal Materials

Quercetin with purity >98% was provided from TLCM (HKUST, Hong Kong). Ginkgo Folium was purchased from commercial sources and morphologically authenticated and qualified by Dr. X. Y. Yang at the Yunnan Institute of Materia Medica according to the Chinese Pharmacopoeia 2015 [15]. Voucher specimens of the herbs were sent to Yannanbaiyao (YNBY) Group Tianzihong Pharmaceutical Co. Ltd. (Kunming, Yunnan, China) to be cataloged.

### 2.2. Preparation of HerboChips

HerboChips were purchased from YNBY Group Tianzihong Pharmaceutical Co. Ltd. Herbal extracts were prepared by extracting 50 g of herb powder with 1 L of ethanol for 3 days, and the ethanoic extract was concentrated to 30 mL. The herbal extracts were then lyophilized and stored in vacuum. Extracts were resuspended in 50/50 water/ethanol and fractionated by a standardized HPLC method in gradient mode with a duration of 96 min (0 min; 0% acetonitrile (ACN) → 96 min; 100% ACN). Fractions were collected according to retention time with each fraction corresponding to 1 min. The chip surfaces were activated, and the epoxy groups were exposed before dotting. The herbal fractions were dotted and fixed on the surface of activated chips by an automatic arrayer (Biodot A101, Shuai Ran Precision, Taiwan). The lyophilized standards were redissolved with DMSO at a concentration of 100 mg/mL as a stock for cell culture studies.

### 2.3. Screening of HerboChips

Biotinylated NGF was prepared using an EZ-Link™ NHS-PEG4-biotinylation kit (Thermo Fisher Scientific, Rockford, IL). About 0.5 mg of NGF (Alomone Labs, Israel) was dissolved in 0.5 mL of phosphate-buffered saline (PBS), mixed with 39 *μ*L of 20 mM biotin solution, and incubated at room temperature for 1 hour. Excess biotin reagent was removed by a desalting column. The labelled NGF was stored at −80°C. About 500 herbal extracts including Ginkgo Folium were selected and screened with the biotinylated NGF probe on the chips. The biotinylated NGF was incubated with HerboChips dotted with different herbal fractions. Streptavidin-Cy5™ (Invitrogen Life Technologies, Carlsbad, CA) was used to detect biotinylated NGF on the HerboChips arrays. The fluorescence signal of streptavidin-Cy5 was measured at 535 nm by a fluorophore microarray scanner (GenePix 4100A, Molecular Devices Corp., Sunnyvale, CA). The fluorescence results were analyzed with GenePix Pro 7 (ver. 7.1.16) software provided by the manufacturer of microarray scanner. Fluorescence intensity higher than 600 was counted as positive. The validation of biotinylated NGF probe using western blat analysis and fluorescence signal imager was performed. Twenty ng of biotinylated NGF was applied to each incubation chamber for 16 hours at room temperature followed by probing with streptavidin-Cy5 for 1 hour at room temperature. Unbound biotinylated NGF probe and streptavidin-Cy5 were removed by repeated rinsing with TBS solution with 0.1% Tween 20.

### 2.4. Enrichment of Identified Fractions

For enrichment of specific fraction, HPLC system with semipreparative column (Dikma, Diamonsil, C18 column, 10.0 × 250 mm^2^, 5 *μ*m) was applied. The injection volume was 20 *μ*L. As soon as the target peak appeared, the peak fraction was collected into another flask separately until the collection wascompleted. Repeating the above procedure described above, the target peaks were completely fished from the total components peaks. The collected fractions were evaporated and redissolved in 1 mL DMSO. A dilution of 1 : 1,000 using culture medium or water was applied for cell assay and analytic measurement.

### 2.5. Chemical Identification by NMR

After enrichment, the unknown fraction, named as D1, was submitted for liquid chromatography with tandem mass spectrometric (LC-MS/MS) analysis and nuclear magnetic resonance (NMR) examination. For LC-MS/MS, the liquid chromatograph is equipped with an Agilent 6410 Triple Quad MS/MS (Agilent, Waldbronn, Germany) and an Eclipse XDBC18 column (2.1 × 100 mm; 3.5 *μ*m particle size). The injection volume was 2 *μ*L. A 20 min linear gradient at flow rate of 0.3 mL/min between solvent A (Milli-Q water, 0.1% formic acid) and solvent B (acetonitrile, 0.1% formic acid) was used. Starting from 30% B, after reaching 70% B, the system returned to 70% A. Retention time of D1 was at 10.5–11.0 min. The MS was operated in negative electron spray ionization mode. A capillary voltage of 3.5 kV and a cone voltage of 10 V were applied. Total ion scanning spectra from *m*/*z* 100 to *m*/*z* 1000 were recorded. For NMR examination, 3 mg of the dried extract was dissolved in 1 mL of methyl sulfoxide-d6 from which 550 *μ*L was drawn and mixed with 50 *μ*L of a D2O solution with 0.2% TSP-d4 and 3 mM sodium azide. TSP-d4 served as an internal standard for NMR, and sodium azide inhibited microbial growth. All particulate materials were removed by centrifugation at 13,000 ×g for 1 min, and the supernatant was transferred to a standard 5 mm NMR tube. NMR spectra were acquired on a Bruker AV 400 MHz NMR spectrometer with a 5 mm PA BBO 400SB BBFO-H-D05 Z-gradient BB observe probe head, operating at 400.13 MHz -NMR frequency at 298 K. Gradient shimming was used to improve the magnetic field homogeneity prior to all acquisition. The NMR spectra of the samples were acquired using a 1D CPMG pulse sequence (RD-90°-*t*1-90°-*tm*-90°-acquire) to generate a spectrum with a reduced residual solvent peak. The experimental time for each sample was around 10 min. All spectra were Fourier-transformed, phase-corrected, and baseline-corrected manually. NMR data was analyzed using MestReNova software.

### 2.6. HPLC Conditions for Ginkgo Folium

The standard solutions of quercetin (1 mM) and extract of Ginkgo Folium, spiked with 0.5 mM quercetin, were prepared. Liquid chromatography was performed on an Agilent 1200 series system (Agilent, Waldbronn, Germany), which was equipped with a degasser, a binary pump, an autosampler, a DAD, and a thermostated column compartment. The herbal extract was separated on a Grace Prevail C-18 column (5 *μ*m id, 250 mm × 4.6 mm). The mobile phase was composed of 0.1% formic acid in acetonitrile (A) and 0.1% formic acid in water (B) using the following gradient program: 0–90 min, linear gradient 25.0–60.0% (A); 90–95 min, linear gradient 60.0–75.0% (A); 95–120 min, linear gradient 75.0–95.0% (A). A preequilibration period of 4 min was used between each run. The flow rate was 0.75 mL/min. The column temperature was 30°C. The injection volume was 100 *μ*L. The UV detector wavelength was set to 330 nm.

### 2.7. Neurite Outgrowth of PC12 Cells

Pheochromocytoma PC12 cells, a cell line derived from rat adrenal medulla, were obtained from American Type Culture Collection (ATCC® CRL-1721™). PC 12 cells were maintained in Dulbecco's modified Eagle's medium supplemented with 6% fetal calf serum, 6% horse serum, 100 units/mL of penicillin, and 100 *μ*g/mL of streptomycin (Invitrogen Life Technologies) in a humidified CO_2_ (7.5%) incubator at 37°C. Culturing media were renewed every 2 to 3 days. Cultured PC12 cells were treated with quercetin or the isolated herbal fraction (D1) and/or NGF for 72 hours, with fresh medium and reagents supplied every 24 hours. A light microscope (Diagnostic Instruments, Sterling Heights, MI) equipped with a phase-contrast condenser, 10x objective lens, and a digital camera (Diagnostic Instruments) was used to capture the images with manual setting. For analyzing the number and length of neurite, approximately 100 cells were counted from at least 10 randomly chosen visual fields for each culture. Using the SPOT software, the cells were then analyzed for number and length of neurite. The cells were scored as differentiated if one or more neurites were longer than the diameter of cell body (i.e., ~ 30 *μ*m).

### 2.8. Phosphorylation of Erk-1/2

Cultured PC 12 cells were starved for 5 hours and then challenged with NGF, quercetin, and extract from Ginkgo Folium for 0, 5, or 10 min. Cells were solubilized in lysis buffer containing 125 mM Tris-hydrochloride (pH 6.8), 4% sodium dodecyl sulfate (SDS), 20% glycerol, and 2% 2-mercaptoethanol and stored frozen at −20°C. Lysates were separated on 8% SDS-polyacrylamide gels and transferred to a nitrocellulose membrane. The membrane was blocked with 5% nonfat milk and then incubated overnight with anti-phospho-Erk-1/2 primary antibodies (1 : 1,000; Cellular Signaling Technology, Danvers, MA), followed by anti-rabbit secondary antibodies (1 : 5,000; Invitrogen Life Technologies) for an hour. The immune complexes were visualized by the enhanced chemiluminescence (ECL) method (GE Healthcare, Chicago, IL). The samples were run on the same gel under strict standardized ECL conditions and the intensities of the bands were compared using an image analyzer and ImageJ 1.48 v software [16].

### 2.9. Statistical Analysis

The protein concentration was measured using the Bradford's method (Bio-Rad Laboratories, Hercules, CA). All data were analyzed using one-way analysis of variance followed by Student's* t*-test (GraphPad Prism 5 (ver 5.01), GraphPad Software, La Jolla CA). Results were classed into three levels of statistical significance: *∗* where *P* < 0.05; *∗∗* where *P* < 0.01, and *∗∗∗* where *P* < 0.001.

## 3. Results

**Figure 1:**
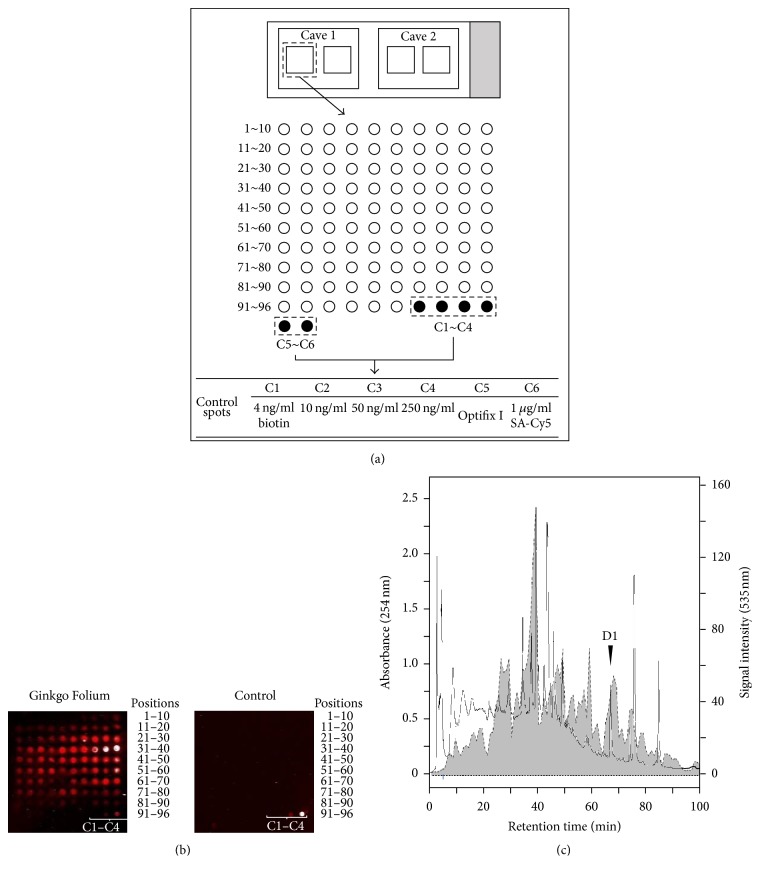
Extract of Ginkgo Folium was fractionated by HPLC and then dotted and fixed on a chip. The chip was incubated with biotinylated protein target (NGF) and then probed with streptavidin-Cy5 before fluorescence detection. (a) Adopted from Huang et al., 2015, showing the design of HerboChip, the quantifier and qualifier controls were included. (b) Scanning result of HerboChip using Ginkgo Folium extract and probed with biotinylated NGF (left) and probed with incubation buffer only (right). Representative scanned images were shown, *n* = 5. (c) Superimposition of the read-out of HerboChip screening and HPLC chromatogram. HPLC chromatograms (solid line) and quantified HerboChip screening read-out (dotted line with shaded area) of the extract of Ginkgo Folium were superimposed. A will resolved peak at retention time ~67 min was labelled as D1 and submitted for further analysis.

**Figure 2:**
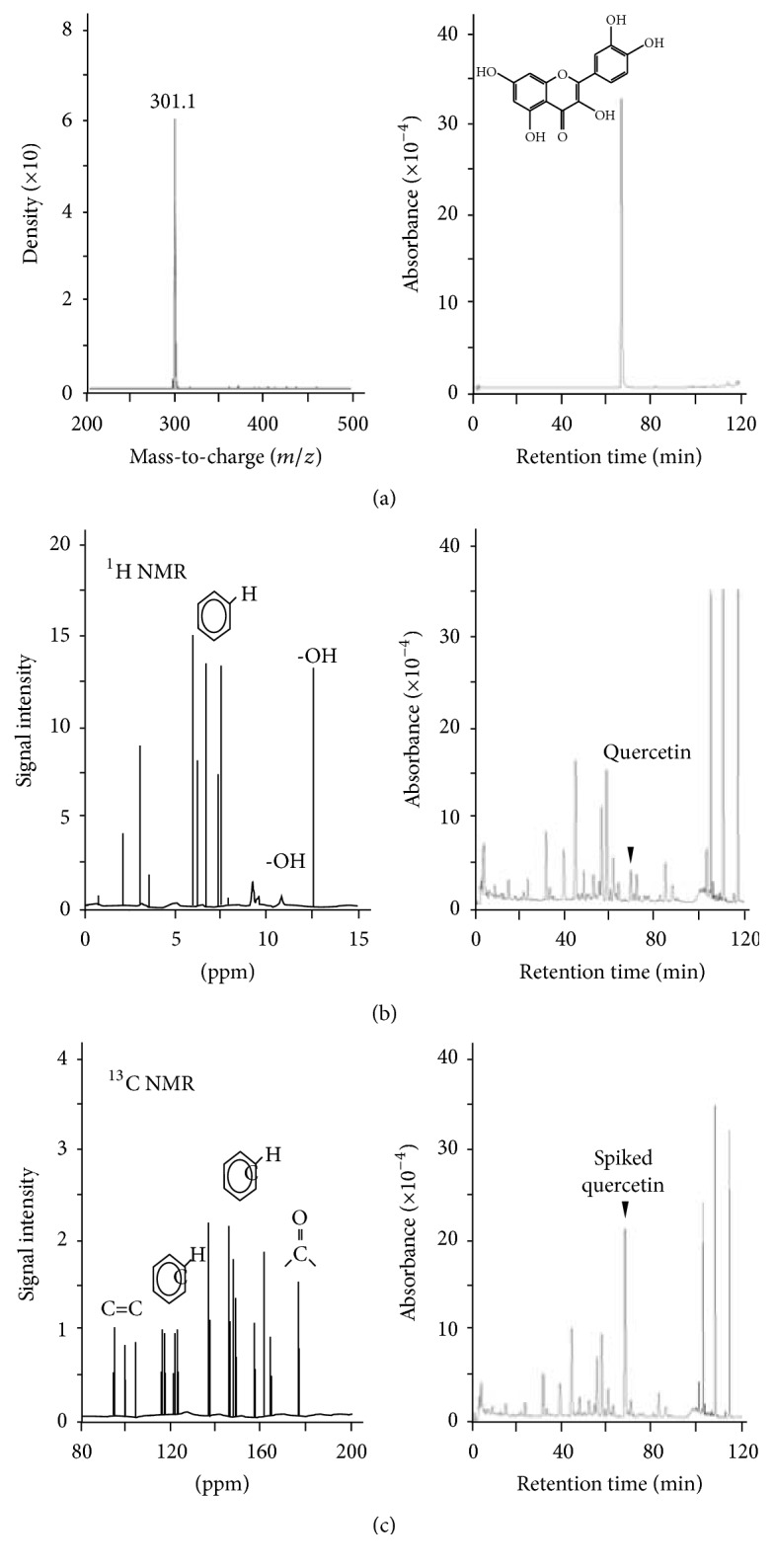
(a) Under full scanning mode, the molecular weight of the enriched fraction D1 was shown by LC-MS/MS. (b) ^1^H-NMR spectra of enriched fraction D1. (c) ^13^C-NMR spectra of enriched fraction D1. Corresponding chemical structures were indicated according to their ppm in different NMR spectrum. The spectrum was submitted to the database, Biological Magnetic Resonance Data Bank (http://www.bmrb.wisc.edu) for characterization. (a) HPLC chromatograms using the HPLC condition described in the section of Materials and Methods were shown: quercetin at 1 mM. Chemical structure of the enriched fraction from the extract of Ginkgo Folium (D1), that is, quercetin, was shown. (b) Extract of Ginkgo Folium (100 mg/mL) and (c) extract of Ginkgo Folium (50 mg/mL) spiked with 0.5 mM quercetin. The position of quercetin was being indicated in (b) and (c).

Around 500 herbal extracts were selected for HerboChips screening. All herbal extracts were screened by HerboChips under standard workflow, as described in our previous report (Figure 1S). The screening probe, NGF, was labelled with biotin. The biotinylated NGF probe was validated by western blot analysis and fluorescence imaging (Figure 2S). The format of array on HerboChips was shown in Figure 1(a), which was adopted from Huang et al. (2015) [17]. From the NGF binding screening, 26 herbal extracts showed positive signals, which was reported by Lee et al. (2016) [12]; however, the identity of NGF binding compounds in those extracts was not revealed. Among the 26 positive-hit herbs, the extract from Ginkgo Folium was the one showing the strongest signaling. A positive signal implied that there was a direct binding between the probe (i.e., NGF) and fractions from extracts. The scanning result was shown for Ginkgo Folium and the blank control (Figure 1(b)). Apart from the control spots dotted with known amount of biotin and SA-Cy5, no positive signal was found in the blank control. As shown in Figure 1, more than one fraction of Ginkgo Folium extracts showed binding with NGF. There were at least two groups of fractions, one from positions 24 to 60 (i.e., elution time 24 min to 60 min after injection of Ginkgo Folium extract) and another one from positions 65 to 70 (i.e., elution time 65 min to 70 min after sample injection). Position 39 and 40 showed the strongest binding signal, as compared to others. The binding signal was quantified by GenePix Pro 7 (ver. 7.1.16) software, and the signal intensity (dotted line) was superimposed with the HPLC chromatograph (solid line) of the extract of Ginkgo Folium (Figure 1(c)). A well-resolved peak (namely D1) was colocalized from both the binding signal and HPLC chromatograph. The fraction D1 was then isolated, enriched, and submitted for further chemical analysis.

The herbal fraction D1 was then submitted for chemical identification. By using LC-MS/MS, D1 fraction was composed of only a single mass at 301.1 *m*/*z* (Figure 2(a)). The compound then underwent ^1^H and ^13^C NMR analysis (Figures 2(b) and 2(c)), and the profiles of NMR were then characterized by submitting to Biological Magnetic Resonance Data Bank (http://www.bmrb.wisc.edu). After analysis, the fraction D1 was identified as quercetin (Figure 2).

**Figure 3:**
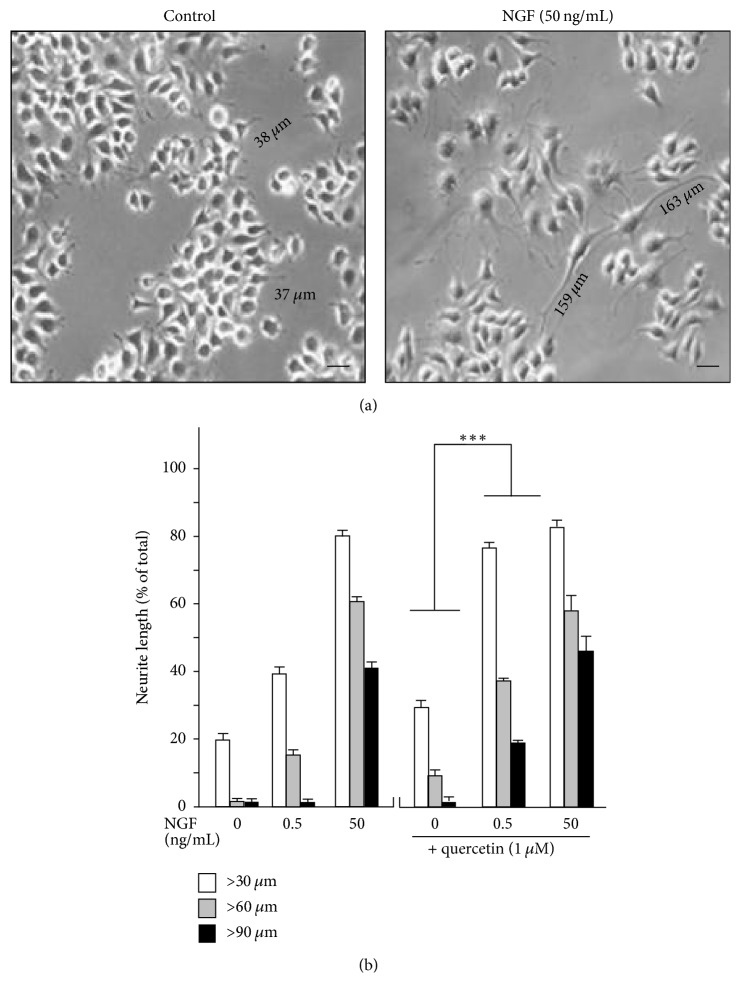
Cultured PC12 cells were treated with control, NGF (0.5 ng/mL or 50 ng/mL), and enriched fraction from the extract of Ginkgo Folium (quercetin) with or without NGF at 0.5 ng/mL for 48 h, and then the neurite outgrowth was examined under microscope. (a) Microscopic image of PC12 cells treated with buffer alone or treated with NGF in 50 ng/mL. Representative images were shown, *n* = 3. Bar = 50 *μ*m. (b) To quantify the differentiation effect, the percentage of differentiated cell numbers and length of neurites were counted by the methods described in Materials and Methods section. Data were expressed as % of cells in 100 counted cells, mean ± SEM,* n* = 3. ^*∗∗∗*^*P* < 0.001 verse control.

**Figure 4:**
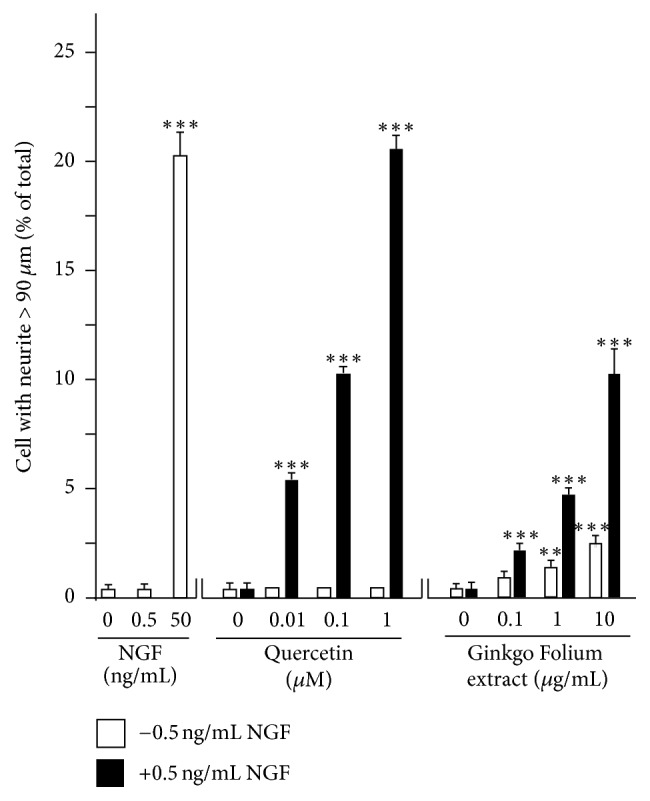
Serum-starved cultured PC12 cells were treated with control, NGF (0.5 ng/mL or 50 ng/mL), or quercetin (0.01–1.0 *μ*M) with or without NGF at 0.5 ng/mL, or the extract of Ginkgo Folium (0.1–10 *μ*g/mL) with or without NGF at 0.5 ng/mL for 48 hours, and then the neurite outgrowth was examined under microscope. To quantify the differentiation effect, the percentage of differentiated cell numbers and length of neurites were counted by the methods described in Material and Methods section. Data were expressed as % of cells in 100 counted cells, Mean ± SEM, *n* = 3. ^*∗∗∗*^*P* < 0.001 verse control.

The role of quercetin, as well as Ginkgo Folium extract, in NGF effect was determined here.  Figure 3(a) shows the control and differentiated PC12 cells in cultures. Having the binding to NGF, we hypothesized that quercetin could interfere NGF normal functions. As a positive control, application of NGF at 50 ng/mL induced robustly PC12 cell to a full differentiated stage (Figures 3(a) and 3(b)). Inclusion of quercetin did not inhibit the NGF-induced neurite outgrowth instead which could slightly increase the neurite growth (Figure 3(b)). To ensure the effect of quercetin could be determined, a low dose of NGF at 0.5 ng/mL not able to induce any growth of neurite was used (Figure 3(b)). Quercetin alone did not increase the neurite out growth significantly; only those having the length of  >30 *μ*m were slightly increased. The coapplication of quercetin and low dose of NGF markedly induced the neurite outgrowth (Figure 3(b)), which suggested a potentiation effect of quercetin in NGF function. By counting the induced neurite of >90 *μ*m in cultured PC 12 cells, quercetin together with low dose of NGF could induce the neurite outgrowth in a dose-dependent manner, and this effect similarly was revealed by using Ginkgo Folium extract (Figure 4).

**Figure 5:**
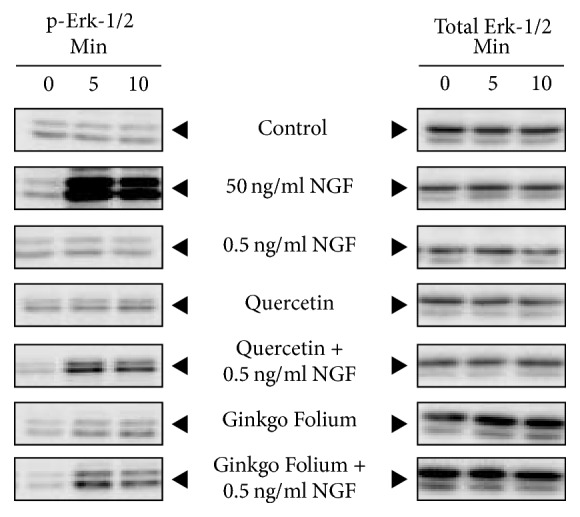
PC12 cells were serum-starved for 3 hours before treatment of control, 0.5 ng/mL, or 50 ng/mL of NGF and quercetin and cotreatment of quercetin with NGF at 0.5 ng/mL. Cell lysates (20 *μ*g) were subjected to western blotting for phosphorylation analysis. Only 50 ng/mL NGF and the cotreated quercetin with 0.5 ng/mL NGF induced phosphorylation of Erk-1/2 (P-Erk-1/2 at ~42 and ~44 kDa) at 5 min after treatments, no notable changes in phosphorylation were observed in other groups, *n* = 4. The representative gels were shown here.

The phosphorylation of Erk-1/2 at 42 and 44 kDa, a downstream effector of NGF signaling, was coherent with neurite outgrowth in cultured PC12 cells in responding to NGF challenge (Figure 5). Notable increment in phosphorylation of Erk-1/2 was observed at 5 min after the cotreatment of 0.5 ng/mL NGF and quercetin. As expected, thers are no obvious changes after the treatment for low level NGF and quercetin alone (Figure 5). In all cases, the total Erk-1/2 did not change.

## 4. Discussion

Quercetin was named in 1857 according to its source, that is, quercetum (oak forest). Quercetin exists in a variety of plants, especially in fruit with color, for example, red onion, berries, and apple (Health remedy 2017). In Chinese medicine, quercetin is also commonly found as major ingredient, for example,* Panax notoginseng *[18] and* Apocynum venetum *[19]. As a dietary flavonoid, the average daily consumption of quercetin could be from 25 to 50 mg. Quercetin is a strong antioxidant and served as scavenger for free radicals. Quercetin can activate both estrogen receptor alpha (ER*α*) and estrogen receptor beta (ER*β*) [20, 21]. Several reports mentioned the beneficial functions of quercetin on nerve system [22]. Those beneficial functions included extension of survivability of neuron by suppressing inflammation [23] and oxidation stress [24, 25]. Here, we showed the potentiation effect of quercetin in NGF-induced neurite outgrowth in cultured PC 12 cells. Our results suggested quercetin might exhibit its potentiation ability via binding of NGF and trigger the downstream signaling in nervous system. Indeed, low expression of NGF is one of the causes for patients suffering from dementia or depression [26]. Thus, this study strengthens the possible usage of quercetin and Ginkgo Folium as health supplements for alleviating dementia or depression.

EGb 761 (Rökan, Tanakan) is a commercial health food product from Ginkgo Folium, which is standardized of having ~ 24% flavone glycosides (primarily quercetin at 5-6%, kaempferol, and isorhamnetin) and 6% terpene lactones (2.8–3.4% ginkgolides A, B, and C and 2.6–3.2% bilobalide). Different lines of evidence have supported the benefits of EGb761 for age-related dementia [27]. In addition to current result of quercetin in NGF function, isorhamnetin, another major flavonoid in Ginkgo Folium, was shown to induce neuronal differentiation [14], synapse formation [28], and secretion of neurotropic factors [29]. Interestingly, isorhamnetin shares similar chemical structure to quercetin.

Characterization of a NGF-bound quercetin from Ginkgo Folium was not the only successful case. HerboChips have also been applied to drug screening for CM that shows binding to tumor necrosis factor (TNF) alpha. The water extract of Andrographis Herba has ability to bind TNF alpha in HerboChips, and this herbal extract is being developed as a product for rheumatoid arthritis [17]. Similarly, our lab is on its way to search herbal extracts binding to vascular endothelial growth factor (VEGF), interleukin 17, *β*-amyloid, and insulin. In view of these routine screening of herbal extracts, the current version of HerboChips was not ideal. A new version of HerboChips must be developed to reach a higher sensitivity. First, HPLC conditions for each herb should be specifically optimized, that is, to ensure a good separation. Second, the content of each pixel in the array should be examined before dotting on the chip, possibly by LC-MS analysis, which could speed up the identification process. Last but not least, an internal position marker, with known migration time in the HPLC analysis, should be included so that the result will not be affected by shifting in the chromatograms.

## 5. Conclusions

The bioactivities of quercetin and Ginkgo Folium on neural functions were illustrated. The potency of neurite development triggered by quercetin or extract from Ginkgo Folium may be due to the binding between NGF and quercetin. Moreover, the current result revealed the potential of transcending the applicability of HerboChips from a screening platform to become a tool for characterizing bioactive compounds on herbs.

## Supplementary Materials

Supplementary Materials 308 work flow of HerboChips screening and validation examination on biotinylated NGF probe are available as Supporting Information. Figure 1S: standard work flow of HerboChips screening. Figure 2S: validation on the efficiencies of biotinylation on nerve grow factor (NGF). Brain lysate and biotinylated NGF were loaded into gel and underwent SDS-PAGE. Images were captured after probing with Cy3 or Cy5 linked streptavidin by a fluorescence imager.

## References

[1] The Henry J. Kaiser Family Foundation Life Expectancy at Birth (in years), by Race/Ethnicity. http://www.kff.org/other/state-indicator/life-expectancy-by-re/.

[2] Alzheimer’s Association What Is Dementia?. http://www.alz.org/what-is-dementia.asp.

[3] Baquero M., Martín N. (2015). Depressive symptoms in neurodegenerative diseases. *World Journal of Clinical Cases*.

[4] Peveler R., Carson A., Rodin G. (2002). Depression in medical patients. *BMJ*.

[5] Swedish Council on Health Technology Assessment Depression treatment for the elderly. http://www.sbu.se/.

[6] Howlett J. R., Paulus M. P. (2013). Decision-making dysfunctions of counterfactuals in depression: who might I have been?. *Frontiers in Psychiatry*.

[7] Richards C. S., O'Hara M. W. (2014). *The Oxford Handbook of Depression and Comorbidity*.

[8] Chen P.-J., Sheen L.-Y. (2011). Gastrodiae rhizoma (tiān má): a review of biological activity and antidepressant mechanisms. *Journal of Traditional and Complementary Medicine*.

[9] Terazaw S. (1986). Practice of Oriental medicine. 1. The importance of Chinese medicine. *[Kangogaku zasshi] The Japanese Journal of Nursing*.

[10] Feng J., Xie P. (2003). The importance and approaches of physicochemical properties analysis of active compounds of traditional Chinese herbs in development of new medicine. *Zhongguo Zhong Yao Za Zhi*.

[11] Shen Z.-Y., Wang W.-J. (2004). Practice has proved the importance of integrating Chinese and Western medicine. *Zhongguo Zhong Xi Yi Jie He Za Zhi*.

[12] Lee P. S. C., Zhang L. M., Yan A. L. (2016). Indication of nerve growth factor binding components from herbal extracts by HerboChip: a platform for drug screening on a chip. *Chinese Medicine*.

[13] Zhu Y., Zhang Z., Zhang M., E. Mais D., Wang M. (2010). High throughput screening for bioactive components from traditional chinese medicine. *Combinatorial Chemistry & High Throughput Screening*.

[14] Xu S. L., Choi R. C. Y., Zhu K. Y. (2012). Isorhamnetin, a flavonol aglycone from Ginkgo biloba L., induces neuronal differentiation of cultured PC12 cells: potentiating the effect of nerve growth factor. *Evidence-Based Complementary and Alternative Medicine*.

[15] Medical BC. Pharmacopoeia of People*ʼ*s Republic of China.

[16] Yan L., Xu S. L., Zhu K. Y. (2015). Optimizing the compatibility of paired-herb in an ancient Chinese herbal decoction Kai-Xin-San in activating neurofilament expression in cultured PC12 cells. *Journal of Ethnopharmacology*.

[17] Huang M., Yao P.-W., Chang M. D.-T. (2015). Identification of anti-inflammatory fractions of Geranium wilfordii using tumor necrosis factor-alpha as a drug target on Herbochip®—an array-based high throughput screening platform. *BMC Complementary and Alternative Medicine*.

[18] Choi R. C., Zhu J. T., Leung K. W. (2010). A flavonol glycoside, isolated from roots of Panax notoginseng, reduce samyloid-beta-induced neurotoxicity in cultured neurons: signaling transduction and drug development for Alzheimer's disease. *Journal of Alzheimers Diseases*.

[19] Cao Y., Zhang X., Fang Y., Ye J. (2001). Determination of active ingredients of apocynum venetum by capillary electrophoresis with electrochemical detection. *Microchimica Acta*.

[20] Maggiolini M., Bonofiglio D., Marrsico S. (2001). Estrogen receptor alpha mediates the proliferative but not the cytotoxic dose-dependent effects of twomajor phytoestrogens on human breast cancer cells. *Molecular Pharmacology*.

[21] van der Woude H., ter Vied M. G. R., Jacobs N., van der Saag P. T., Murk A. J., Rietjens I. M. C. M. (2005). The stimulation of cell proliferation by quercetin is mediated by the estrogen receptor. *Molecular Nutrition Food Research*.

[22] Elumalai P., Lakshmi S. (2016). Role of quercetin benefits in neurodegeneration. *The Benefits of Natural Products for Neurodegenerative Diseases*.

[23] Zhang Y., Yi B., Ma J. (2015). Quercetin promotes neuronal and behavioral recovery by suppressing inflammatory response and apoptosis in a rat model of intracerebral hemorrhage. *Neurochemical Research*.

[24] Chakraborty J., Rajamma U., Jana N., Mohanakumar K. P. (2015). Quercetin improves the activity of the ubiquitin-proteasomal system in 150Q mutated huntingtin-expressing cells but exerts detrimental effects on neuronal survivability. *Journal of Neuroscience Research*.

[25] Li Y. L., Guo H., Zhao Y. Q., Zhang J. W. (2017). Quercetin protects neuronal cells from oxidative stress and cognitive degradation induced by amyloid *β*-peptide treatment. *Molecular Medicine Reports*.

[26] Hefti F., Armanini M. P., Beck K. D. (1996). Development of neurotrophic factor therapy for Alzheimer's disease. *CIBA Foundation Symposia*.

[27] DeFeudis F. V. (2002). Effects of Ginkgo biloba extract (EGb 761) on gene expression: Possible relevance to neurological disorders and age-associated cognitive impairment. *Drug Development Research*.

[28] Xu S. L., Zhu K. Y., Bi C. W. C. (2013). Flavonoids induce the expression of synaptic proteins, synaptotagmin, and postsynaptic density protein-95 in cultured rat cortical neuron. *Planta Medica*.

[29] Xu S. L., Bi C. W., Choi R. C. (2013). Flavonoids induce the synthesis and secretion of neurotrophic factors in cultured rat astrocytes: a signaling response mediated by estrogen receptor. *Evidence-Based Complementary and Alternative Medicine*.

