# Supplementary material for: Quercetin Potentiates the NGF-Induced Effects in Cultured PC 12 Cells: Identification by HerboChips Showing a Binding with NGF
Source: Evid Based Complement Alternat Med. 2018 Feb 28;2018:1502457. doi: 10.1155/2018/1502457 (PMC5850895; doi:10.1155/2018/1502457)
Supplement: Supplementary Materials — 308 work flow of HerboChips screening and validation examination on biotinylated NGF probe are available as Supporting Information. Figure 1S: standard work flow of HerboChips screening. Figure 2S: validation on the efficiencies of biotinylation on nerve grow factor (NGF). Brain lysate and biotinylated NGF were loaded into gel and underwent SDS-PAGE. Images were captured after probing with Cy3 or Cy5 linked streptavidin by a fluorescence imager. [file 1502457.f1.docx]

**Supporting information**

**Quercetin Potentiates the NGF-induced Effects in Cultured PC 12 Cells: Identification by HerboChips^®^ Showing a Binding with NGF**

Gallant KL Chan^1,2^, Winnie W Hu^2^ , Zoey X Zheng^1^, Yan Lin^1,2^, Caroline Wang^2^, M Huang^3^, XY Yang^3^, Karl WK Tsim^1,2^, Tina TX Dong^1,2^

^1^Shenzhen Research Institute, The Hong Kong University of Science and Technology, Shenzhen, 518057, China

^2^Division of Life Science and Center for Chinese Medicine, The Hong Kong University of Science and Technology, Hong Kong

^3^YNBY Lab INC., No. 51, Xi-Ba Rd., Kunming, Yunnan Province, PR China 650032.

**Correspondence**

Dr. Tina Dong, Shenzhen Research Institute, The Hong Kong University of Science and Technology, Shenzhen, 518057, China. Email: [botina@ust.hk](mailto:botina@ust.hk) Phone: +852 2358 7318 Fax +852 2358 7323

**
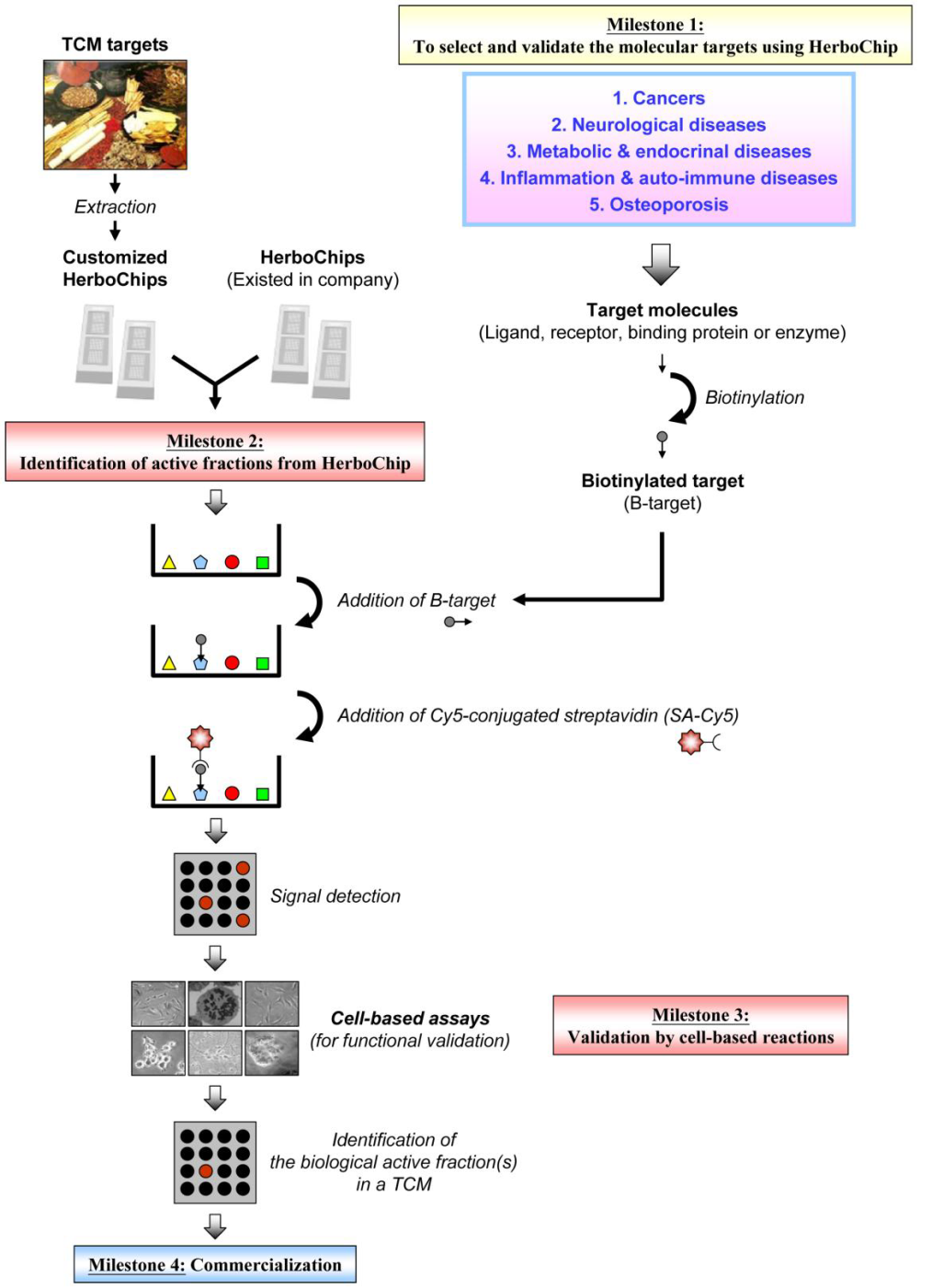
**

**Fig. 1S** Standard work flow of HerboChips screening


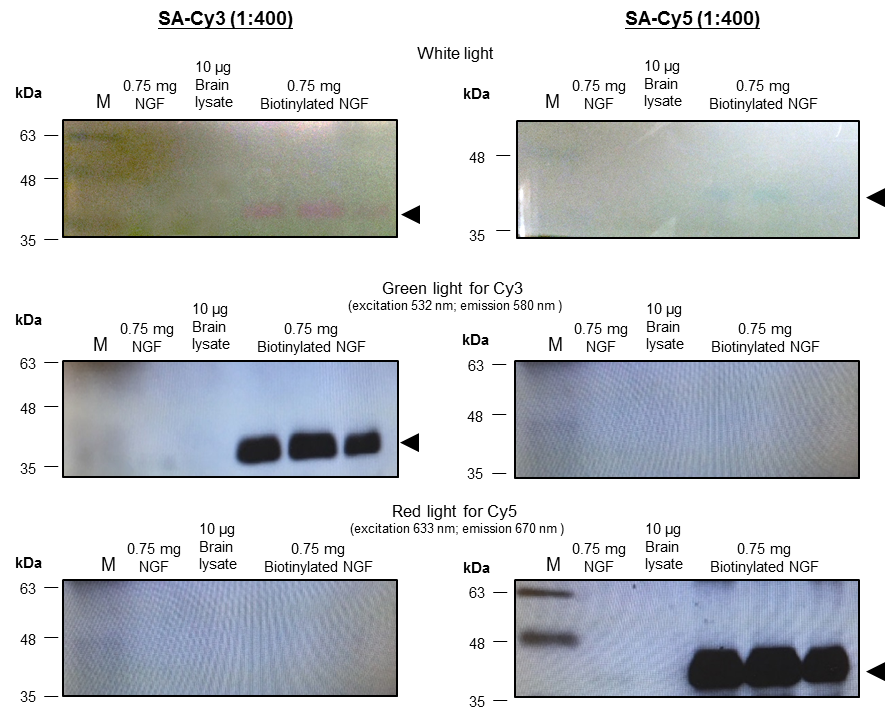


**Fig. 2S** Validation on the efficiencies of biotinylation on nerve grow factor NGF). Brain lysate and biotinylated NGF were loaded into gel and underwent SDS-PAGE, Image were captured after probing with Cy3 or Cy5 linked streptavidin by a fluorescence imager.
